# Active case detection for malaria elimination: a survey among Asia Pacific countries

**DOI:** 10.1186/1475-2875-12-358

**Published:** 2013-10-09

**Authors:** Cara Smith Gueye, Kelly C Sanders, Gawrie NL Galappaththy, Christina Rundi, Tashi Tobgay, Siv Sovannaroth, Qi Gao, Asik Surya, Garib D Thakur, Mario Baquilod, Won-ja Lee, Alby Bobogare, Sarath L Deniyage, Wichai Satimai, George Taleo, Nguyen M Hung, Chris Cotter, Michelle S Hsiang, Lasse S Vestergaard, Roly D Gosling

**Affiliations:** 1 Global Health Group, University of California, San Francisco, 50 Beale Street, Suite 1200, San Francisco, CA USA; 2 World Health Organization, Myanmar, PO Box 14 11182, Yangon, Myanmar; 3 Sabah State Health Department, Ministry of Health, Malaysia, Level 3, Federal House 88814, Kota Kinabalu, Sabah Malaysia; 4 Vector-Borne Disease Control Programme, Ministry of Health, Royal Government of Bhutan, Post Box 191, Gelephu, Bhutan; 5 National Center for Parasitology, Entomology and Malaria Control, Ministry of Health Cambodia, #372, Monivong Bld, Phnom Penh, Cambodia; 6 Jiangsu Institute of Parasitic Diseases, Meiyuan, Wuxi, Jiangsu 214064 People’s Republic of China; 7 National Malaria Control Program, Ministry of Health Republic of Indonesia, Ditjen PP & PL, Gd.C Lt.1, Jln. Percetakan Negara No.29, Jakarta, Indonesia; 8 Government of Nepal Ministry of Health and Population Department of Health Services, Epidemiology & Disease Control Division, Teku, Kathmandu Nepal; 9 Department of Health, Philippines, Infectious Disease Office, National Center for Disease Prevention and Control, San Lazaro Compound, Sta.Cruz, Manila Philippines; 10 Divisions of Malaria and Parasitic Diseases, National Institute of Health, Korea Centers for Disease Control and Prevention; Osong Health Technology Administration Complex187, Osong Sangmyeong 2 ro, Gangoe-myeon, Cheongwon-gun, ChungCheongbuk-Do 363-951 Republic of Korea; 11 Ministry of Health Solomon Islands, PO Box 349, Honiara, Solomon Islands; 12 Sri Lanka Anti-Malaria Campaign, Ministry of Health Sri Lanka, No: 555/5, Public Health Complex, Elvitigala Mawatha, Narahenpita, Colombo – 05 Sri Lanka; 13 Bureau of Vector Borne Disease, Department of Disease Control, Ministry of Public Health, Nonthaburi 11000 Thailand; 14 National Vector Borne Disease Control Program, Ministry of Health, Vanuatu, Port Vila Vanuatu; 15 15National Institute for Malariology, Parasitology and Entomology, Ministry of Health, Vietnam, Luong The Vinh street, BC 10200, TuLiem District Hanoi, Vietnam; 16 Department of Pediatrics, University of California, San Francisco, 500 Parnassus Ave, MU411E San Francisco, CA USA; 17 Western Pacific Regional Office, World Health Organization, P.O. Box 2932, UN Avenue, 1000 Manila, Philippines

**Keywords:** Malaria elimination, Surveillance, Active case detection, Case investigation

## Abstract

**Background:**

Moving from malaria control to elimination requires national malaria control programmes to implement strategies to detect both symptomatic and asymptomatic cases in the community. In order to do this, malaria elimination programmes follow up malaria cases reported by health facilities to carry out case investigations that will determine the origin of the infection, whether it has been imported or is due to local malaria transmission. If necessary, the malaria programme will also carry out active surveillance to find additional malaria cases in the locality to prevent further transmission. To understand current practices and share information on malaria elimination strategies, a survey specifically addressing country policies on case investigation and reactive case detection was carried out among fourteen countries of the Asia Pacific Malaria Elimination Network (APMEN).

**Methods:**

A questionnaire was distributed to the malaria control programme managers amongst 14 countries in the Asia Pacific who have national or sub-national malaria elimination goals.

**Results:**

Results indicate that there are a wide variety of case investigation and active case detection activities employed by the 13 countries that responded to the survey. All respondents report conducting case investigation as part of surveillance activities. More than half of these countries conduct investigations for each case. Over half aim to accomplish the investigation within one to two days of a case report. Programmes collect a broad array of demographic data during investigation procedures and definitions for imported cases are varied across respondents. Some countries report intra-national (from a different province or district) importation while others report only international importation (from a different country). Reactive case detection in respondent countries is defined as screening households within a pre-determined radius in order to identify other locally acquired infections, whether symptomatic or asymptomatic. Respondents report that reactive case detection can be triggered in different ways, in some cases with only a single case report and in others if a defined threshold of multiple cases occurs. The spatial range of screening conducted varies from a certain number of households to an entire administrative unit (e g, village). Some countries target symptomatic people whereas others target all people in order to detect asymptomatic infections. The majority of respondent programmes collect a range of information from those screened for malaria, similar to the range of information collected during case investigation.

**Conclusion:**

Case investigation and reactive case detection are implemented in the malaria elimination programmes in the Asia Pacific, however practices vary widely from country to country. There is little evidence available to support countries in deciding which methods to maintain, change or adopt for improved effectiveness and efficiency. The development and use of common evaluation metrics for these activities will allow malaria programmes to assess performance and results of resource-intensive surveillance measures and may benefit other countries that are considering implementing these activities.

## Background

Over the last decade, major investments in malaria control have led to substantial decreases in the global burden of disease [1,2]. Today, 99 countries are malaria free, with an additional 34 currently eliminating malaria [1,3]. Malaria elimination is gaining global political support, and for many countries, national malaria elimination is no longer a question of if, but rather when it will occur [4].

The transition from malaria control to elimination is complex, requiring a shift in strategy and the introduction of new activities that must be tailored to a country’s individual context. A robust surveillance system for the detection of symptomatic and asymptomatic cases, in addition to notification, reporting and investigation of all malaria infections is crucial [1,4-6]. Detailed case investigations are particularly important, as they allow malaria control programmes to determine the origin (indigenous or imported) of a case, and conduct appropriate activities in response [7].

Traditionally, countries have used passive case detection (PCD) to capture cases, relying on symptomatic patients to present to a health facility for diagnosis and treatment [8]. It has been well documented, however, that asymptomatic and subclinical or sub-patent infections are common and contribute substantially to ongoing transmission [5,8-10]. These individuals do not have malaria symptoms, do not seek treatment, and remain infected for long periods and are, therefore, a source for onward transmission without their knowledge. A control programme may conduct active case detection (ACD) to seek out infection and residual parasite carriers, a strategy recommended by the World Health Organization (WHO) that can be particularly useful for capturing asymptomatic infections [7]. Malaria infections in all transmission settings tend to be clustered geographically in a focus, defined by WHO as a 'defined, circumscribed locality situated in a currently or former malarious area containing the epidemiological factors necessary for malaria transmission’ [7]. At a finer resolution, infections cluster into microfoci or “hotspots”, which can be comprised of individuals, households or groups of households that maintain ongoing transmission in a community [11]. In some low transmission settings, malaria is clustered in demographic groups or specific high-risk populations, known as “hot” populations, or “hot-pops”, that may carry infection from their work place to their villages or may be at higher risk of infection because of behavioural factors [6]. If infections are clustered in hotspots, then geographically based active surveillance strategies may be effective at preventing further malaria transmission and reducing transmission towards zero.

ACD is defined by WHO as the 'detection by health workers of malaria infections at community and household level in population groups that are considered to be at high risk. ACD can be conducted as fever screening followed by parasitological examination of all febrile patients or as parasitological examination of the target population without prior screening’ [7].

Countries engage in a wide variety of activities that they consider to fall within the scope of their ACD strategy, and may include proactive case detection (PACD) and reactive case detection (RACD) [12,13]. PACD consists of targeted or mass screening to search for cases in the community, which may include screening to find cases that are symptomatic or asymptomatic without the trigger of passively detected cases [14,15]. RACD is an active surveillance method that is triggered by passively detected cases and involves screening households or individuals within a specified area, typically a pre-determined radius around a locally acquired case, with the goal of preventing further malaria transmission by identifying additional infections, symptomatic or asymptomatic [8,16]. Many countries have or are currently implementing PACD and/or RACD to achieve and maintain malaria elimination [8,14,15,17]. Despite these efforts, there is a lack of guidance on how, when and where to employ ACD, and limited evidence exists on the effectiveness of ACD as a strategy to halt ongoing transmission [6,17].

This study aims to describe and compare case investigation and ACD strategies and activities currently employed in partner countries of the Asia Pacific Malaria Elimination Network (APMEN), a group of 14 countries (Bhutan, Cambodia, China, Democratic People’s Republic of Korea, Indonesia, Malaysia, Nepal, Philippines, Republic of Korea, Solomon Islands, Sri Lanka, Thailand, Vanuatu, and Vietnam) in the Asia Pacific region. A major goal of APMEN is to provide a platform to gather and share national malaria control programme experiences and strategies employed for malaria elimination, such as ACD. For Solomon Islands and Vanuatu, the strategies and activities described in this study only reflect those undertaken in the elimination provinces, as the two countries presently have sub-national malaria elimination goals.

## Methods

A survey on ACD activities was developed in collaboration with national malaria control programmes and partner organizations of APMEN. The survey aimed to gather information on strategies employed by national malaria control programmes to investigate and detect malaria cases, specifically related to index case investigation and RACD. Questions pertained to: standard operating procedures (SOPs), classification of cases as local or imported, RACD methods, screening measures used in response to local and/or imported cases, monitoring and supervision of programme staff, and additional measures (e g, vector control and entomological surveillance) conducted in response to cases. Countries were also asked to provide national level SOPs or other available materials to supplement survey responses.

Definitions of case investigation, active and reactive case detection were provided to survey respondents as part of the survey form. The definitions of case investigation and active case detection were adapted from the definition provided by WHO [18]. Case investigation was defined as 'gathering enough information to allow classification of a malaria case by origin of infection. It includes, but is not limited to, administration of a standardized questionnaire to a person diagnosed with malaria infection’. Active case detection was defined as 'the operation carried out by surveillance agents who visit every locality in a defined area at regular intervals (usually monthly during the transmission season), in order to enquire for fever cases through individual house visits, and to test for malaria (and treat if positive) each suspected person so discovered’. Of note, a revised WHO definition of active case detection was issued while the survey was underway, from involving screening symptomatic individuals only to including screening both asymptomatic and symptomatic individuals [7]. The definition used in the survey for reactive case detection was adapted from Moonen *et al*. [16], and was described as 'triggered whenever a case is identified by passive case detection…and will involve visiting the household of the locally acquired case, screening family members, and screening neighbors within a defined radius’.

The survey was piloted in November 2011 with three APMEN collaborating country partners and one partner organization. Revisions were made according to the results of the pilot survey. In December 2011, the survey was distributed to country representatives of each APMEN country partner, of which there were 11 at the time. The survey was then distributed to the three new APMEN country partners in October 2012. Follow-up, including questions on missing or unclear text answers, was conducted in May 2013.

Quantitative and qualitative data were entered into an Excel database, and a descriptive analysis of quantitative survey questions was conducted in STATA IC, version 12. Qualitative data were analysed in Excel.

## Results

The national malaria control programmes of 14 APMEN countries were invited to participate in the ACD survey, of which 13 responded to the survey. Some countries responded to all questions and some failed to respond to certain questions or entire components of the survey.

Table 1 describes the participating countries and their respective nationally reported cases from 2010 and the country’s total population [19-21]. Each country’s annual parasite index (API), or the number of reported malaria cases per 1,000 risk population per year, was reported by country programmes in the survey. Most respondent programmes (eight of ten) in the survey described case investigation and RACD practices that programmes aim to conduct universally throughout the country. Two respondents, Solomon Islands and Vanuatu, reported that they conduct the activities described in the survey only in designated malaria elimination provinces. The Solomon Islands has a goal of eliminating malaria from Temotu and Isabel Provinces by 2014 [13]. Tafea Province in Vanuatu is targeted for elimination by 2014 [22].

**Table 1:** Survey respondents and malaria indicators

**Respondent**	**2010 Total cases**	**2010 Total population**	**2010 Annual parasite index**
BHUTAN	436	695,819	0.63
CAMBODIA	56,217	14,138,255	4.07
CHINA	7,855	1,341,335,152	0.01
INDONESIA	465,764	237,641,326	1.75
MALAYSIA	6,650	28,250,000	0.24
NEPAL	3,115	21,249,567	0.14
PHILIPPINES	19,644	92,337,852	0.21
REPUBLIC OF KOREA	1,721	48,183,584	0.04
SOLOMON ISLANDS	35,373	538,148	65.73
SRI LANKA	684	20,859,949	0.03
THAILAND	32,480	69,122,234	0.47
VANUATU	4,017	239,651	16.76
VIETNAM	54,297	87,202,813	0.62

Annual Parasite Index - Cases per 1,000 risk population, as reported by countries.

### Protocols and reporting

Of the 12 countries that responded to the questions on protocols and reporting, most (nine) have developed a SOP for case investigation and/or additional screening in the community, and ten of 12 use a written case investigation report form when conducting investigations.

### Case investigation

Thirteen of the respondents reported that they conduct case investigation as part of their surveillance activities. Over half (seven of 13) respondents reported that they conduct case investigation for all cases. Three investigate between 26 to 99% of cases, and three reported conducting case investigation for up to 25% of all cases. The survey did not ask for information on what occurs for cases that are not investigated. The event that triggers a case investigation was described by 11 of 13 countries as a case reported to either the national or peripheral level. For one of the two remaining countries, case investigation is triggered when there are “multiple cases from one village, or an individual case reported from an area typically without malaria.” The second country did not respond to this question.

Over half (seven of 13) of countries reported that case investigation begins between one to two days after a case is reported, while five countries have a time period of three to seven days and one country has no defined time period.

### Personnel and supervision

All countries (13) reported that there is a specific person in the malaria control programme who is tasked with conducting case investigation. Nearly all programmes (11) reported that these officers are trained in case investigation techniques, and 11 countries reported that personnel conducting investigations are periodically supervised by managers. This supervision ranges from each investigation, on a quarterly basis, once per year, or on an irregular basis.

### Activities conducted and information collected

During case investigation there are several actions taken and types of information collected from the index case (Table 2). Nearly all countries (12) visit the index case, supervise treatment (12), follow up on adherence to treatment (ten), check on malaria prevention measures used by the index case (12), and educate the index case on malaria risk factors and prevention (ten). Most programmes map the location of the index case (nine), and five of these countries use geographical information system (GIS) to make the maps.

**Table 2:** Actions taken and information collected during case investigation

	**BTN**	**KHM**	**CHN**	**IDN**	**MYS**	**NPL**	**PHL**	**KOR**	**SLB**	**LKA**	**THA**	**VUT**	**VNM**
Conduct case investigations	**●**	●	●	●	●	●	●	●	●	●	●	●	●
Visit the index case	●	●	●	●	●		●	●	●	●	●	●	●
Retest index case	●		●	●				●	●		●	●	●
Supervise treatment	●	●	●	●	●		●	●	●	●	●	●	●
Follow up on adherence to treatment	●	●		●	●		●	●		●	●	●	●
Check prevention measures used	●	●		●	●	●	●	●	●	●	●	●	●
Educate individual on risk factors & prevention	●	●		●	●		●	●	●	●	●	●	
Collect information on anti-malarial medicines in use		●		●	●	●		●		●	●		
Map location of residence of index case		●	●	●	●		●	●		●	●	●	
Collect information on travel history	●	●	●	●	●	●	●	●	●	●	●	●	●
*Travel within district to malarious areas*	●	●	●	●	●		●	●	●	●	●	●	
*Travel outside district*	●	●	●	●	●		●	●	●	●	●	●	●
*Travel outside country*	●	●	●	●	●	●	●	●	●	●	●	●	●
*Number of days upon return from travel until symptom onset*	●	●		●	●		●	●	●	●	●	●	●
Collect information on recent contact with travelers/ immigrants			●	●	●	●	●	●		●	●	●	
Collect information on history of G6PD deficiency				●			●				●		

For Solomon Islands and Vanuatu, the activities apply only to subnational elimination provinces.

**Legend:** Country abbreviations: Bhutan: BTN; Cambodia: KHM; China: CHN; Indonesia: IDN; Malaysia: MYS; Nepal: NPL; Philippines: PHL; Republic of Korea: KOR; Solomon Islands: SLB; Sri Lanka: LKA; Thailand: THA; Vanuatu: VUT; Vietnam: VNM.

All respondents (13) reported that their programme collects information from the index case on travel history, with the majority of respondents collecting information on travel within (11) or outside (12) the district of residence, or outside of the country (13) (Table 2). Nine programmes participating in the survey collect information on whether the index case has had any recent contact with travellers or immigrants. Only three countries gather patient history of glucose-6-phosphate dehydrogenase (G6PD) deficiency, an inherited blood disorder prevalent in many malaria-endemic areas.

### Determination of a case as imported or indigenous

Out of the 13 respondents, nine countries defined imported cases as those originating in another country. The remaining countries, including the countries that have subnational elimination goals, reported importation as those infections occurring within the country but from a different province, district or other administrative unit. Ten countries collected data and reported on this type of intra-country importation (e g, from different districts). Table 3 describes the type of information that is taken into consideration when determining an imported case.

**Table 3:** Information used in determination of an imported case

	**BTN**	**KHM**	**CHN**	**IDN**	**MYS**	**NPL**	**PHL**	**KOR**	**SLB**	**LKA**	**THA**	**VUT**	**VNM**
Travel location	●	●		●	●	●	●	●		●	●	●	●
Geographic location and timing of onset of symptoms	●	●		●	●		●	●					●
Malaria endemicity of local residence	●			●	●		●			●			

The categories are not mutually exclusive.

For Solomon Islands and Vanuatu, the activities apply only to subnational elimination provinces.

**Legend:** Country abbreviations: Bhutan: BTN; Cambodia: KHM; China: CHN; Indonesia: IDN; Malaysia: MYS; Nepal: NPL; Philippines: PHL; Republic of Korea: KOR; Solomon Islands: SLB; Sri Lanka: LKA; Thailand: THA; Vanuatu: VUT; Vietnam: VNM.

### Reactive case detection

When asked whether programmes conduct RACD, 12 of 13 countries conduct this type of screening. Survey respondents reported that RACD was triggered in three different ways, and some country programmes reported several triggers for their programme: for eight countries, every indigenous case is a trigger (e g, one case identified through passive case detection considered to be local); all imported cases irrespective of duration of stay are a trigger for five countries; and one country conducts screening around imported cases if they have stayed more than a certain number of days in country (range of one to 30 days).

Table 4 depicts the threshold number of infections, identified through passive case detection that triggers RACD in individual countries, regardless of whether the case is imported or local. Other triggers for additional screening include: if there is a need to measure the API in a given area, if a person with symptoms or a positive test result occurs among travellers with whom the index case is identified, or if there is an unusual increase in cases in a community in a particular time interval, indicating a possible outbreak.

**Table 4:** Triggers for reactive case detection

	**BTN**	**KHM**	**CHN**	**IDN**	**MYS**	**NPL**	**PHL**	**KOR**	**SLB**	**LKA**	**THA**	**VUT**	**VNM**
Single confirmed case	●	●	●		●			●	●	●	●		
> 1 confirmed case within specified radius				●		●	●					●	
Other threshold of confirmed cases													●

A “single confirmed case” refers to a case identified through passive case detection, or an index case.

For Solomon Islands and Vanuatu, the activities apply only to subnational elimination provinces.

**Legend:** Country abbreviations: Bhutan: BTN; Cambodia: KHM; China: CHN; Indonesia: IDN; Malaysia: MYS; Nepal: NPL; Philippines: PHL; Republic of Korea: KOR; Solomon Islands: SLB; Sri Lanka: LKA; Thailand: THA; Vanuatu: VUT; Vietnam: VNM.

Populations targeted for RACD vary across the surveyed countries. Five countries reported conducting screening of only symptomatic people within the household of the index case, while six countries screen all (both symptomatic and asymptomatic) household members. Regarding the screening of neighbours of the index case household, five countries reported that they conduct screening of symptomatic neighbours in addition to the household of the index case while six countries screen both symptomatic and asymptomatic neighbours. Four respondents reported screening symptomatic people within a certain political boundary, while two screen all people within a political boundary. Five countries reported screening asymptomatic people during reactive case detection and two countries reported screening only symptomatic people. The radius screened from the index household for all countries ranged from 0.5 to 2.5 km.

When conducting RACD, several methods of diagnosis are used and some are used in combination with others for diagnosis confirmation and speciation (results not mutually exclusive). All (13) respondents reported using microscopy, seven use rapid diagnostic tests (RDT), five use polymerase chain reaction (PCR), two use clinical diagnosis, and one uses serology (Table 5).

**Table 5:** Diagnostic methods used during reactive case detection

	**BTN**	**KHM**	**CHN**	**IDN**	**MYS**	**NPL**	**PHL**	**KOR**	**SLB**	**LKA**	**THA**	**VUT**	**VNM**
Clinical diagnosis						●		●					
Microscopy	●	●	●	●	●	●	●	●	●	●	●	●	●
Rapid diagnostic tests		●		●		●		●		●	●		●
Polymerase chain reaction		●	●	●				●			●		
Serology								●					

For Solomon Islands and Vanuatu, the activities apply only to subnational elimination provinces.

**Legend:** Country abbreviations: Bhutan: BTN; Cambodia: KHM; China: CHN; Indonesia: IDN; Malaysia: MYS; Nepal: NPL; Philippines: PHL; Republic of Korea: KOR; Solomon Islands: SLB; Sri Lanka: LKA; Thailand: THA; Vanuatu: VUT; Vietnam: VNM.

### Epidemiological information collected during reactive case detection

During RACD, survey respondents collect information across two different groups: those screened that have a positive test or all persons screened whether or not they have a positive test. General results indicate that there is a variety of information collected, whether in positives or in all screened. See Table 6 for details about the countries that collect information for both groups. Out of 12 respondents, ten programmes collect information on the length of time spent at the current residence. All countries (the 13 survey participants) collect data on occupation during screening and nine collect information on their place of work. Eleven countries of 13 collect information on the travel history to malaria-endemic areas, and nine countries of 12 collect information on recent contact with travellers or immigrants. Three countries of 12 ask about history of G6PD deficiency. Ten countries of 13 map the residences of either the positive cases or of all those that are screened.

**Table 6:** Actions taken and information collected during reactive case detection

	**BTN**	**KHM**	**CHN**	**IDN**	**MYS**	**NPL**	**PHL**	**KOR**	**SLB**	**LKA**	**THA**	**VUT**	**VNM**
Conducts RACD	●	●	●	●	●	●	●	●	●	●	●		●
Collect data on length of time at current residence		▪	▪	▪	▪	▪	x	▪		▪	▪	▪	
Collect information on occupation	x	x	▪	▪	▪	▪	▪	▪	▪	▪	▪	▪	x
Collect information on place of work		x	▪	▪	▪	▪	x	▪		▪	▪		
Collect information on travel history to malarious areas	x	x	▪	▪	▪	▪	▪	▪		▪	▪	▪	
Collect information on recent contact with travelers or immigrants			▪	▪	▪	▪	x	▪		▪	▪	▪	
Collect information on history of G6PD deficiency				▪	x		x						
Map locations of residences of those screened		▪	▪	▪	▪	▪	x	▪		x	▪	▪	

For Solomon Islands and Vanuatu, the activities apply only to subnational elimination provinces.

**Legend:** Squares represent activities undertaken for test-positive subjects only; x represent activities undertaken for all screened individuals.

Country abbreviations: Bhutan: BTN; Cambodia: KHM; China: CHN; Indonesia: IDN; Malaysia: MYS; Nepal: NPL; Philippines: PHL; Republic of Korea: KOR; Solomon Islands: SLB; Sri Lanka: LKA; Thailand: THA; Vanuatu: VUT; Vietnam: VNM.

### Additional measures

As part of case investigation practices, country programmes implement several types of activities, including vector control, entomological surveillance, or health education. Of the ten respondents who completed this section of the survey, all reported conducting indoor residual spraying (IRS) and a form of health education and behaviour change (information, education communication (IEC) or behaviour change communication (BCC)) as part of the response measures during case investigation or RACD. Six of the eight respondents who answered these questions reported distribution of insecticide-treated bed nets (ITNs) or long-lasting insecticide-treated bed nets (LLINs) as part of these practices. Nine of 11 countries which answered this question use some form of larval control measures as part of a case investigation process. Twelve respondents reported conducting entomological surveillance as part of investigation procedures, three of which do so in all cases. Lastly, targeted mass drug administration (MDA) is conducted as part of case investigation by one country (out of 12 respondents).

## Discussion

Survey results indicate that systems and procedures for case investigation and RACD are widely in place in the malaria elimination programmes of the APMEN partner countries. Thirteen out of the 14 partner countries responded to the survey and all of the respondent countries carried out case investigation, while 12 reported using RACD. Across respondents, the strategies employed varied, especially in regard to RACD, with different people screened (symptomatic *versus* asymptomatic) and the number of people screened (household only *versus* the whole village or a 2.5 km radius). Which of these RACD approaches is effective at preventing and reducing transmission is not known. Better understanding of which strategy is most effective is critical as they are human resource intensive.

Index case investigation practices varied, including the proportion of cases for which case investigation occurred. Most respondents (seven) reported following WHO guidelines to conduct case investigation within one to two days of a case being detected [7]. Many countries also reported collecting broad demographic data during case investigation, most of which are recommended by the WHO, such as: current address, length of time at that address, occupation and place of work, recent travel history, and recent contact with known malaria cases [7]. Although most respondents reported following case investigation data collection guidelines, there is an array of parameters that each programme chooses to assess, and likely reflect tailoring of investigations to local conditions and programme capacity. In some countries, supervision and management of case investigation occurs regularly, also likely related to programme capacity.

How programmes determine the origin of a case also differed across the respondent countries. Although “imported” cases are typically defined as a case originating in another endemic country, several respondents – including those with sub-national elimination strategies as those with national elimination strategies – reported defining cases as imported if they originate from another endemic district or province within the country itself. Programmes seem to prioritize local context when defining importation.

RACD in Asia Pacific countries involves screening households within a specified area, typically a pre-determined radius, around a locally acquired case with the goal of identifying other infections that might be symptomatic or asymptomatic. This process is similar to that found in other countries [14,17,23]. However, there is diversity in the strategies and activities used for RACD. Survey results from the Asia Pacific countries show that RACD can be triggered with one case or a defined threshold of multiple index cases, depending on local incidence and malaria control programme resources. Several countries reported screening symptomatic members of the index case household, while others screen all residents of the household. Recent evidence regarding the importance and frequency of asymptomatic infection in low transmission settings suggests that screening symptomatic people alone will not effectively and rapidly reduce malaria transmission [5,10]. More evidence on the degree of clustering in the settings of APMEN partner countries is needed to support decision-making on how far to screen around an index household. Some countries reported screening all households within a specific administrative unit (e g, village) or a certain radius around the index case, a maximum of 2.5 km. The decision on how wide to screen is based on the theoretical dispersal of vectors and the operational capacity of the programme. However, it should be noted that it is operationally challenging to screen large numbers of households [24], as the radius around the index household increases the area to be covered increases by the square of the radius.

Most programmes collect a range of information from those screened, including information on residence, occupation, travel history, contact with travellers or immigrants, and other details. Different diagnostic methods (clinical diagnosis, microscopy, RDT, PCR, serology) are used to screen during RACD. There are particular challenges in diagnosing cases in areas with a high proportion of sub-patent or sub-microscopic cases [25]. Several countries are now using PCR confirmation in addition to other diagnostic methods, however, it is not known how routinely it is used in these countries and little evidence exists regarding which diagnostic methods are most effective in the setting of the Asia Pacific [17]. The response to a case or an outbreak should be well coordinated and include vector control and public health messaging components. This was reflected in the survey, as many countries include additional vector control, entomological surveillance and/or health education activities [13]. Although the results of this study show widespread use of RACD, there is a dearth of evidence to guide countries on the effectiveness of these activities.

The variation in case investigation and RACD practices across Asia Pacific countries illustrates the need for further research and informed guidance. In particular, the variation in triggers used for RACD and disparate target areas for screening - either the number of households or a radius - indicate that there remain gaps in knowledge to support optimal identification of malaria infections in the community and to identify the most effective and efficient way to capture them. A starting point to address these gaps would be the collection of standard data on both case investigation and RACD activities. Important metrics to assess are case investigation and RACD coverage rates, number of people screened and completeness of geographical coverage during RACD as well as timeliness of these activities. The survey showed that many programmes do not systematically collect robust data on these activities. Thus there is a need to establish these standardized metrics to monitor and evaluate programme effectiveness. The actual choice of metrics should be established through country programme and stakeholder consultation and integrated into routine surveillance data collection, taking into account the varying contexts in which programmes operate, namely funding constraints, epidemiology, geography and others.

### Limitations

Respondents provided information on the type and scale of activities and data collected, based on their programme strategies and policies. It is likely that many strategies as described are likely not undertaken routinely, as their execution depends on availability of funding, human resources and other constraints. However, an assessment of whether the description in the survey results matches the on the ground reality was not within the scope of this survey, and would best be measured through an observational approach. Gathering more detailed information on why countries selected a particular approach or strategy likewise was not within the scope of the survey, although it is assumed that most base their strategies on WHO surveillance guidelines.

The countries with sub-national elimination strategies – Solomon Islands and Vanuatu – reported solely on the surveillance strategies and activities undertaken in the elimination provinces. The survey did not attempt to collect information on the activities in the control provinces thus a comparison of the two is not possible.

## Conclusion

As countries progress towards national or sub-national elimination, malaria programmes should devote staff time to investigating cases and conducting reactive surveillance activities to seek out remaining reservoirs of infection. It is critical for programmes to achieve high coverage of the population, ultimately reaching every case to determine the origin of the infection and if the infection has spread, and mount a response when necessary. Knowing the origin of each case is a critical component of an elimination campaign; it allows a programme to identify pockets or specific populations that contribute to ongoing transmission, and preserves programme resources by avoiding RACD work in areas where it may be unnecessary. Where RACD is necessary, it should be carried out in the most effective way.

Currently there are no standard metrics in use by country programmes for case investigation and RACD. The development and use of common metrics for these activities will allow the programmes to assess performance and results of resource-intensive surveillance measures and may benefit countries that considering implementing these activities in other parts of the world. There is much to learn from countries that are well on the way towards malaria elimination. This APMEN survey is a small step in distilling and disseminating this information.

## Abbreviations

ACD: Active case detection; API: Annual parasite index; APMEN: Asia Pacific Malaria Elimination Network (APMEN); BCC: Behaviour change communication; GIS: Geographical information systems; G6PD: Glucose-6-phosphate dehydrogenase; IEC: Information education, communication; IRS: Indoor residual spraying; ITN: Insecticide-treated bed net; LLIN: Long-lasting insecticide-treated bed nets; MDA: Mass drug administration; PACD: Proactive case detection; PCD: Passive case detection; PCR: Polymerase chain reaction; RACD: Reactive case detection; RDT: Rapid diagnostic test; SOP: Standard operating procedure; WHO: World health organization.

## Competing interests

The authors declare that they have no competing interests.

## Authors’ contributions

CSG, CR, GNLG, KCS, LV, MSH and RG developed the research topic. CSG, CR, GNLG, KCS, LV and RG developed the survey instrument. CSG and KCS conducted the survey. AB, AS, CR, GG, GQ, GDT, GT, MB, MSH, NH, RG, SD, SS, TT, WL and WS provided data and feedback on the manuscript. CC, CSG, and KCS analysed data. CSG and KCS drafted the manuscript. LV is a staff member of the World Health Organization and alone is responsible for the views expressed in this publication, and they do not necessarily represent the decisions or policies of the World Health Organization. All authors read and approved the final version of the manuscript.
